# Starch content changes and metabolism-related gene regulation of Chinese cabbage synergistically induced by *Plasmodiophora brassicae* infection

**DOI:** 10.1093/hr/uhab071

**Published:** 2022-01-19

**Authors:** Yinbo Ma, Su Ryun Choi, Yu Wang, Sushil Satish Chhapekar, Xue Zhang, Yingjun Wang, Xueying Zhang, Meiyu Zhu, Di Liu, Zhennan Zuo, Xinyu Yan, Caixia Gan, Di Zhao, Yue Liang, Wenxing Pang, Yong Pyo Lim

**Affiliations:** 1 College of Horticulture, Shenyang Agricultural University, Shenyang 110866, China; 2 Molecular Genetics and Genomics Laboratory, Department of Horticulture, Chungnam National University, Daejeon 305-764, Republic of Korea; 3 Cash Crops Research Institute, Hubei Academy of Agricultural Sciences, Hubei Key Laboratory of Vegetable Germplasm Enhancement and Genetic Improvement, Wuhan 430070, China; 4 Analytical and Testing Center, Shenyang Agricultural University, Shenyang 110866, China; 5 College of Plant Protection, Shenyang Agricultural University, Shenyang 110866, China

## Abstract

Clubroot is one of the major diseases adversely affecting Chinese cabbage (*Brassica rapa*) yield and quality. To precisely characterize the *Plasmodiophora brassicae* infection of Chinese cabbage, we developed a dual fluorescent staining method for simultaneously examining the pathogen, cell structures, and starch grains. The number of starch (amylopectin) grains increased in *B. rapa* roots infected by *P. brassicae*, especially from 14 to 21 days after inoculation. Therefore, the expression levels of 38 core starch metabolism genes were investigated by quantitative real-time PCR. Most genes related to starch synthesis were up-regulated at 7 days after *P. brassicae* inoculation, whereas the expression levels of starch degradation-related genes were increased at 14 days after inoculation. Then, genes encoding the core enzymes involved in starch metabolism were investigated by assessing their chromosomal distributions, structures, duplication events, and synteny among *Brassica* species. Genome comparisons indicated that 38 non-redundant genes belonging to six core gene families related to starch metabolism are highly conserved among *Arabidopsis thaliana, B. rapa*, *Brassica nigra*, and *Brassica oleracea*. Previous genome sequencing projects have revealed that *P. brassicae* obtained host nutrients by manipulating plant metabolism. Starch may serve as a carbon source for *P. brassicae* colonization, as indicated by histological observations and transcriptomic analysis. Results of this study may elucidate the evolution and expression of core starch metabolism genes and provide researchers with novel insights into the pathogenesis of clubroot in *B. rapa*.

## Introduction

Clubroot, caused by a soil-borne biotrophic pathogen, *Plasmodiophora brassicae*, is a serious global threat to field-grown *Brassica* species. After infection by *P. brassicae*, plant roots often form galls, whereas the aboveground parts turn yellow, which adversely affects the final yield. Clubroot is very difficult to prevent and control because *P. brassicae* resting spores remain viable in the soil for >17 years in the absence of host plants [1]. Until recently, there was a lack of effective methods for controlling clubroot on *Brassica* plants. Clubroot management has been based on the following two main strategies: preventing the pathogen from entering pathogen-free fields, and developing resistant cultivars [2, 3]. Therefore, research on clubroot has mainly focused on the identification of disease resistance genes and breeding for new disease-resistant varieties. There has been relatively little research on the pathogenicity of *P. brassicae.* Although the life cycle of the pathogen has been reported [4–6], how it obtains nutrients and other components from the host is not comprehensively understood. In the family Brassicaceae, 330 genera and 3700 species are possible hosts of *P. brassicae*, including some cruciferous weeds (stinkweed and shepherd’s purse) [7, 8]. There is a need to investigate why the Brassicaceae family is suitable for the invasion and proliferation of *P. brassicae* and what genomic commonalities need to be understood.

Cytological research is important for clarifying the *P. brassicae* life cycle because it can elucidate the changes in various substances that occur during the infection of plants by *P. brassicae*. A histochemical analysis of plant tissues can elucidate the precise life phases of *P. brassicae*. Many staining techniques have been developed to identify pathogens, host structures, and changes in host cells during *P. brassicae* infections, including those utilizing methylene blue, astra blue, safranin, azure II, toluidine blue, 4′,6-diamidino-2-phenylindole, basic fuchsin, and Nile red alone or combined with other stains [9, 10]. Most of these methods can differentiate between *P. brassicae* and host cells in the late infection stages. However, during the early infection stage, it is difficult to distinguish between plasmodia and starch grains in host cells [11, 12]. Accordingly, a staining method applicable to the early infection stage needs to be developed to enable more thorough examinations of the complete infection process.

Starch serves as a transient and long-term carbohydrate reserve in plants and other eukaryotic organisms [13]. Starch metabolism provides the carbon and energy required for many physiological processes that are mainly associated with nocturnal, stress, and germination events. Previous research on potato and rapeseed revealed that the starch content is higher in plants infected by fungi than in uninfected control plants [14–16]. Transcriptome analyses demonstrated that the expression levels of genes encoding some core enzymes in the starch synthesis pathway are up-regulated in poplar and watermelon plants infected by *Botryosphaeria dothidea* and *Botrytis cinerea*, respectively [17, 18]. Previous studies indicated that the starch content increases in *Arabidopsis thaliana* roots infected by *P. brassicae*, but there has been no further research on the effects of *P. brassicae* on host plant starch metabolism [19, 20]. Previous studies on *Brassica rapa* also indicated that infected cells and clubroot galls contain more starch grains than healthy cells and roots [21, 22]. Genes encoding starch metabolism enzymes have been studied mainly in *A. thaliana* [23, 24], potato [25, 26], and rice [27, 28]. Little is known about these core enzyme genes and their roles in *B. rapa* infected by *P. brassicae*. The core genes involved in starch metabolism are crucial for *P. brassicae*’s potential for manipulating plant metabolism to take nutrients from the host [29–31]*.* Fortunately, the available genome information can be used to identify the starch metabolism genes in *Brassica* crops (http://brassicadb.cn/). The relevant genomic distribution, structure, and duplication of these core genes as well as the syntenic analysis of *B. rapa* (AA genome), *Brassica nigra* (BB genome), and *Brassica oleracea* (CC genome) are important to reveal the commonalities of *Brassica* crops susceptible to *P. brassicae* [32].

In this study, we developed a dual fluorescent staining method useful for investigating the cytological characteristics of host cells and *P. brassicae* in infected and uninfected Chinese cabbage roots. The amylopectin level increased in *B. rapa* roots infected by *P. brassicae*. The expression profiles of the core enzyme genes related to starch metabolism were analyzed to reveal the *P. brassicae*-induced starch metabolism regulatory network in *B. rapa*. We also identified core genes encoding the following enzymes participating in starch metabolism in *A. thaliana*, *B. rapa*, *B. nigra*, and *B. oleracea*: ADP-glucose pyrophosphorylase (AGPase), starch branching enzyme (SBE), starch synthase (SS), starch debranching enzyme (DBE), α-amylase (AMY), and β-amylase (BAM)*.* The chromosomal distribution, structure, duplication, and synteny of these identified genes were investigated. The results of this study may provide researchers with a comprehensive atlas of the cytological characteristics and starch metabolism during *P. brassicae* infection of *B. rapa*. The presented data will be useful for future studies aimed at increasing our understanding of the regulation of starch metabolism and the evolutionary divergence of these starch metabolism genes related to *P. brassicae* infections of the Brassicaceae family*.*

## Results

### Development of a dual fluorescent staining method for detecting *P. brassicae*

**Figure 1:**
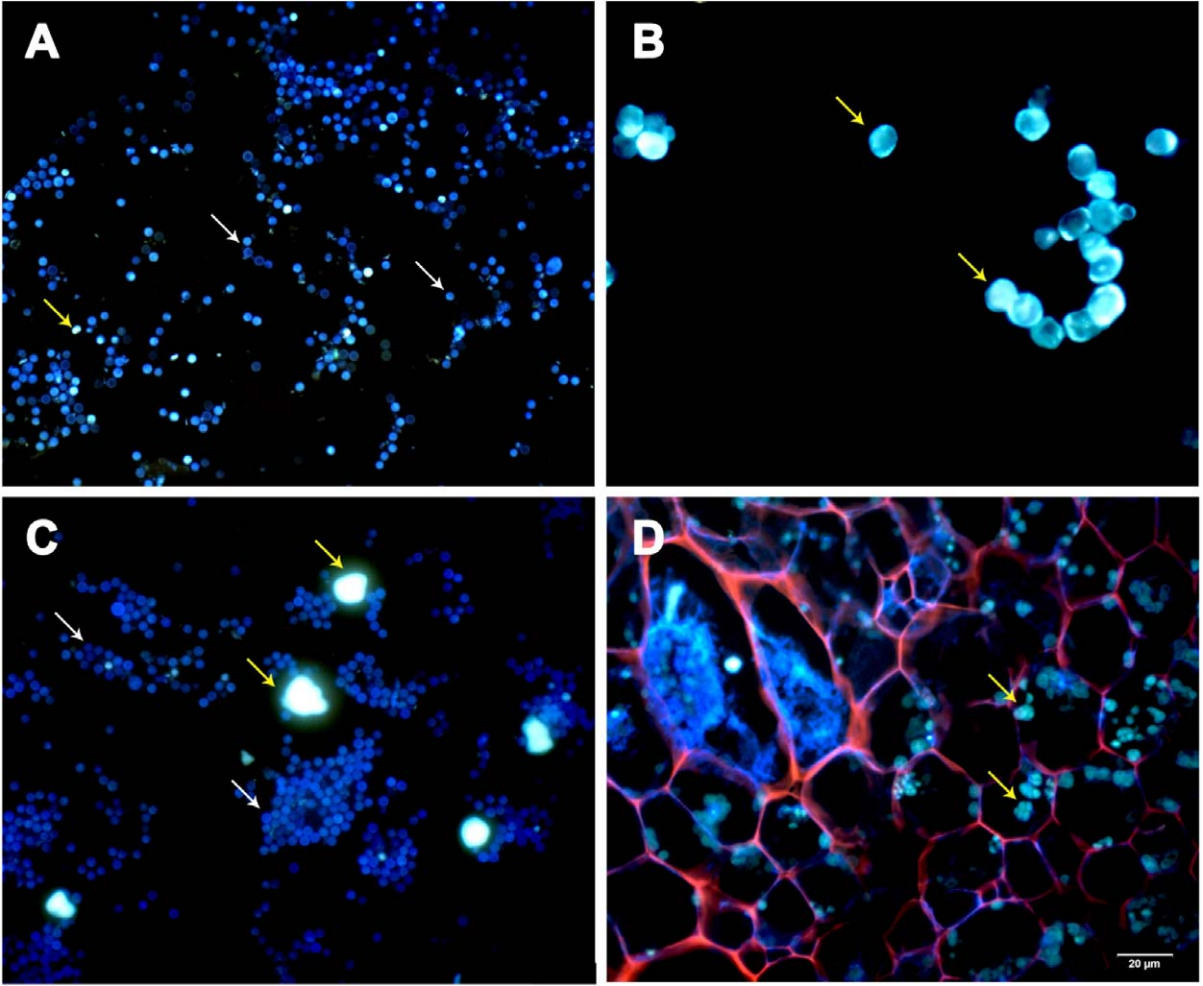
Dual fluorescent staining of resting spores, amylopectin from potato, and Chinese cabbage root cross sections using aniline blue and Nile red. **a** Resting spores of *P. brassicae* isolated from Chinese cabbage. **b** Amylopectin from potato. **c** Mixture of resting spores and amylopectin. **d** Cross sections of Chinese cabbage roots 3 weeks after inoculation with *P. brassicae* (10 replicates). The white and yellow arrows indicate *P. brassicae* spores and starch grains, respectively. The red arrow indicates the plasmodium in the infected cell. Scale bars represent 20 μm.

**Figure 2:**
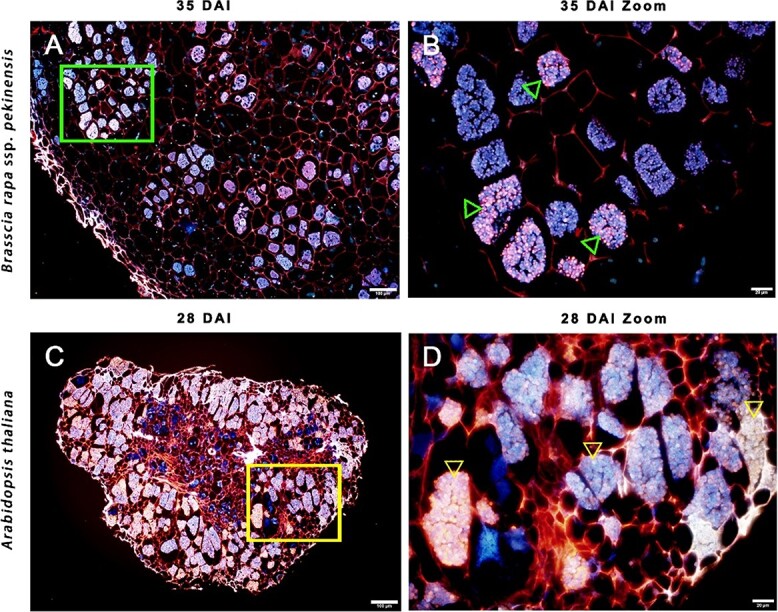
Miroscope observation of *P. brassicae* resting spore forming stages in *B. rapa* and *A. thaliana* stained with aniline blue and Nile red. Green and yellow triangles indicate *P. brassicae* resting spores in *B. rapa* and *A. thaliana*, respectively. Scale bars represent 100 μm (**a** and **c**) or 10 μm (**b** and **d**).

To better identify *P. brassicae*, cell structures, and starch grains, we developed a dual fluorescent staining method using aniline blue and Nile red. The isolated *P. brassicae* resting spores were stained blue (Fig. 1a and c), whereas the amylopectin from potato was stained bright green (Fig. 1b and c). The Chinese cabbage root cell walls and plasmodium were stained red and blue, respectively (Fig. 1d). The starch grains were stained bright green in the roots of Chinese cabbage infected by *P. brassicae*. Interestingly, different colors, such as gold, yellowish brown, purple pink, and white, were observed in *P. brassicae* resting spore-forming stages in *B. rapa* and *A. thaliana* (Fig. 2).

### Starch accumulation initiated by *P. brassicae* infection

**Figure 3:**
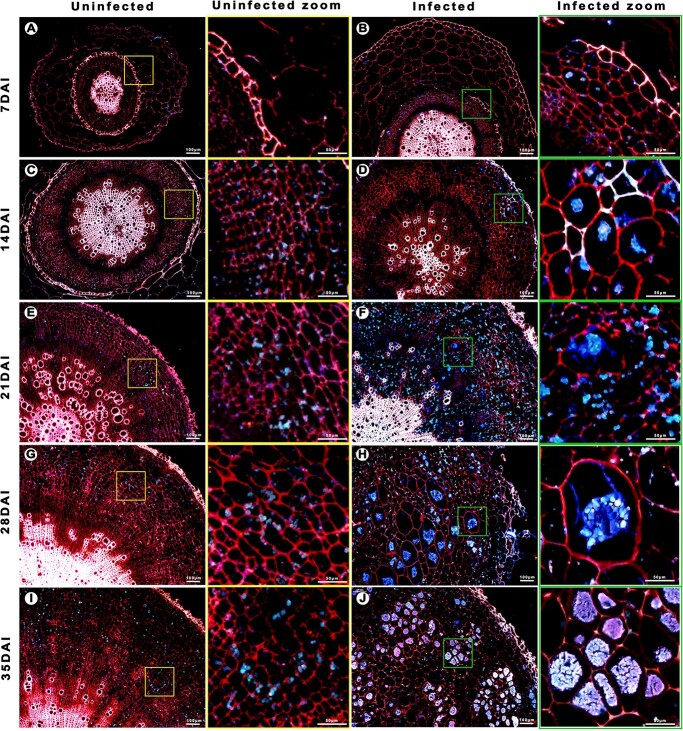
Clubroot disease development in Chinese cabbage ‘BJN3-1’ roots infected by *P. brassicae* and uninfected roots. **a**, **c**, **e**, **g**, and **i** Representative 10-μm cross-sections of samples collected 7, 14, 21, 28, and 35 days after mock inoculation, respectively. **b**, **d**, **f**, **h**, and **j** Representative 10-μm cross-sections of samples collected 7, 14, 21, 28, and 35 days after inoculation, respectively.

Starch accumulation was investigated during infection of *B. rapa* by *P. brassicae* using the developed dual fluorescent staining method. Microscope observation showed consistent results in all 10 biological replicates of root cross-sections (Fig. 3). Specifically, *P. brassicae* plasmodia were detected in infected Chinese cabbage roots from 14 days after inoculation (DAI), whereas the plasmodia were undetectable in uninfected roots (Fig. 3). Infected parenchyma cells were irregular and were 2–16 times larger than the corresponding cells in uninfected plants. Starch grains were observed both in uninfected and infected roots from 14 DAI (Fig. 3). Starch grain accumulation peaked at 21 DAI in infected roots. The number of starch grains decreased at 28 and 35 DAI, whereas the amount of *P. brassicae* plasmodia increased. Resting spores were first detected at 35 DAI.

### Qualitative and quantification of starch in roots

**Figure 4:**
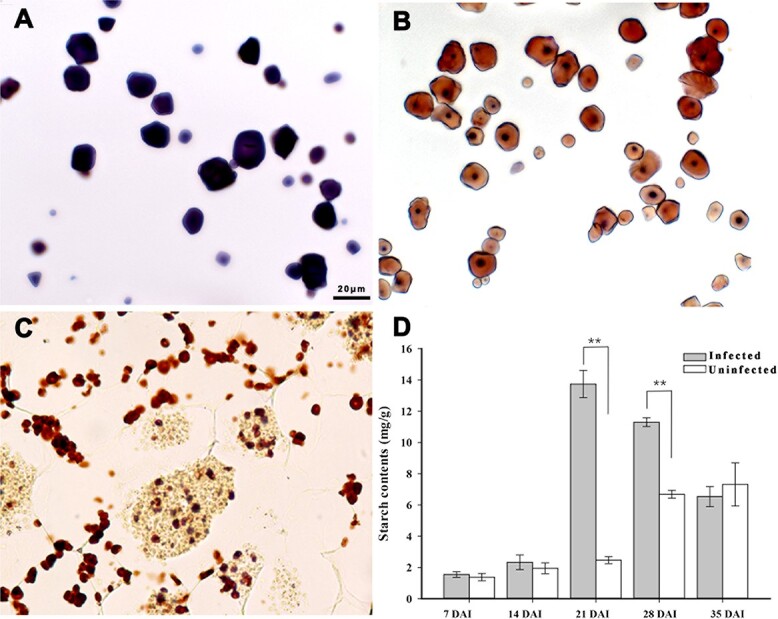
Iodine staining of amylose and amylopectin from potato and root cross-sections 3 weeks after inoculation of *B. rapa* with *P. brassicae*. **a** Amylose. **b** Amylopectin. **c** Root-cross sections of *P. brassicae*-infected *B. rapa* (10 replicates). **d** Total starch contents of infected and uninfected Chinese cabbage roots. Asterisks indicate significant differences between the ‘infected’ and ‘uninfected’ lines based in a *t*-test (independent): **P < .01.

Amylose and amylopectin from potato were stained blue and brown, respectively. Starch in the infected Chinese cabbage roots at 21 DAI was stained brown (Fig. 4), implying that amylopectin was the starch component in Chinese cabbage roots infected by *P. brassicae*.

The root total starch content gradually increased from 7 to 35 DAI in the uninfected plants, whereas it increased from 7 to 21 DAI and then decreased from 28 to 35 DAI in the infected plants. The total starch content in the root differed significantly between the infected and uninfected plants at 21 and 28 DAI (Fig. 4). The highest starch content was detected in infected roots at 21 DAI (i.e. 5 times higher than in uninfected roots).

### Identification and classification of starch metabolism genes

By analyzing the domains of 30 members of six gene families related to the starch metabolic pathway in *A. thaliana*, we identified domains in the following starch synthesis pathway enzymes: all AGPases contained only one NTP_transferase domain (Pfam: PF00483); SS contained the glyco_transf_5 domain (Pfam: PF08323); the debranching enzyme (ISA) included the CBM_48 (Pfam: PF02922) and α-amylase (Pfam: PF00128) domains; and all SBEs had the α-amylase_C (Pfam: PF02806) and α-amylase (Pfam: PF00128) domains. Of the enzymes in the starch degradation pathway, AMY contained the α-amylase_C2 (Pfam: PF07821) and α-amylase (PF00128) domains, whereas β-amylase (BAM) contained the glyco_hydro_14 domain (Pfam: PF01373) (Supplementary Table S1).

We identified 38 non-redundant genes related to the starch metabolic pathway in the *B. rapa* genome (Table 1; Supplementary Table S1). According to their roles related to starch synthesis and degradation, the candidate starch-related genes were divided into six subfamilies and their relationships were visualized in a phylogenetic tree (Supplementary Fig. S1). The gene classification was consistent with the classification of the corresponding *A. thaliana* genes. Gene names were assigned according to their domain types and their orthologs in *A. thaliana*. Regarding the starch synthesis pathway in *B. rapa*, 21 non-redundant genes were identified, which encoded eight AGPases, six SSs, four ISAs, and three SBEs. The remaining 17 genes, which encoded 14 BAMs and 3 AMYs, were associated with the starch degradation pathway.

**Table 1:** Summary of core genes related to starch metabolism in *B. rapa*, *A. thaliana*, *B. nigra*, and *B. oleracea*.

Pathway	Predicted type	*A. thaliana*	*B. rapa*	*B. nigra*	*B. oleracea*
Starch synthesis	ADP-glucose pyrophosphorylase (AGPase)	6	8	8	8
	Starch synthase (SS)	6	6	6	7
	Starch branching enzyme (SBE)	3	3	3	3
	Debranching enzyme (ISA)	3	4	4	4
Starch degradation	α-Amylase (Amy)	3	3	4	3
	β-Amylase (BAM)	9	14	13	18
Total		30	38	38	43

### Expression analysis of starch metabolism genes in response to a *P. brassicae* infection

**Figure 5:**
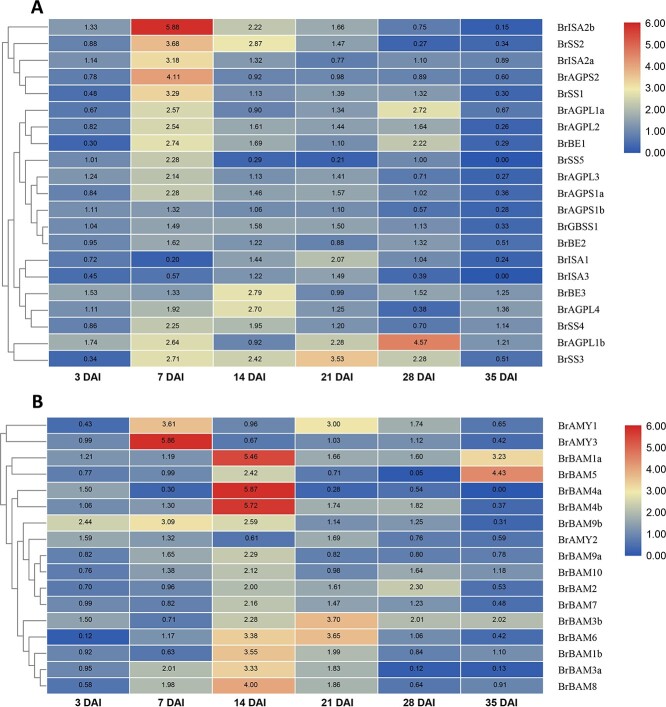
Relative expression of starch metabolism genes in *B. rapa*. **a** Genes related to the starch synthesis pathway. **b** Genes related to the starch degradation pathway.

To analyze the starch metabolism gene expression profiles in *B. rapa* in response to *P. brassicae* infection, we collected Chinese cabbage ‘BJN3-1’ root samples at 3, 7, 14, 21, 28, and 35 DAI with the pathogen or water for a quantitative real-time PCR (qRT–PCR) assay. The differences in the gene expression profiles suggested that the gene families were differentially regulated during *P. brassicae* infection of Chinese cabbage. The expression levels of the majority of the genes related to the starch synthesis pathway were up-regulated at 7 DAI. Specifically, *BrAGPS2* and *BrISA2b* expression levels were significantly up-regulated at 7 DAI and gradually decreased from 14 to 35 DAI (Fig. 5a). Thus, these two starch metabolism genes may be important for *B. rapa* responses to *P. brassicae.* The AMY and BAM genes associated with the starch degradation pathway were differentially expressed. For example, *BrAMY1* and *BrAMY3* were highly expressed during the initial infection period (i.e. up to 7 DAI), after which they gradually decreased (Fig. 5b). In contrast, most BAM genes were highly expressed at 14 DAI. More specifically, *BrBAM4a*, *BrBAM4b*, and *BrBAM8* expression levels gradually increased from 3 to 14 DAI and then decreased from 14 to 35 DAI, implying that they are important genes during the *P. brassicae* infection of *B. rapa* (Fig. 5b). Additionally, genes related to the starch synthesis pathway were actively expressed at 7 DAI. However, at 14 DAI, genes related to the starch degradation pathway were activated. In summary, the expression of ISA, AGPS, and BAM gene family members was correlated with starch contents of infected root. Thus, the ISA, AGPS, and BAM families were focused on in further analyses, while genomic information on the remaining gene families (SS, SBE, AMY) are mentioned in Supplementary Tables S1–S5 and Supplementary Figs S1–S3.

### Genomic distribution, characteristics, and structures of starch metabolism genes

Knowledge of the starch metabolism genes and their gene structure and characteristics in *B. rapa* (AA genome), *B. nigra* (BB genome), and *B. oleracea* (CC genome) is important to reveal the commonalities of *Brassica* crops suitable for the invasion and reproduction of *P. brassicae*. We therefore used the same approach to analyze the starch metabolism genes in *B. nigra* and *B. oleracea*, which are genetically closely related to *B. rapa*. A total of 38 (*B. nigra*) and 43 (*B. oleracea*) candidate genes from six core gene families related to starch metabolism were identified. Genomic distribution analysis demonstrated that 30 genes (100%) in *A. thaliana*, 38 genes (100%) in *B. rapa*, 36 genes (94.74%) in *B. nigra*, and 42 genes (95.45%) in *B. oleracea* mapped randomly and unevenly on the chromosomes (Supplementary Fig. S2, Supplementary Table S1). The remaining starch metabolism genes were distributed on unanchored contigs or scaffolds.

**Figure 6:**
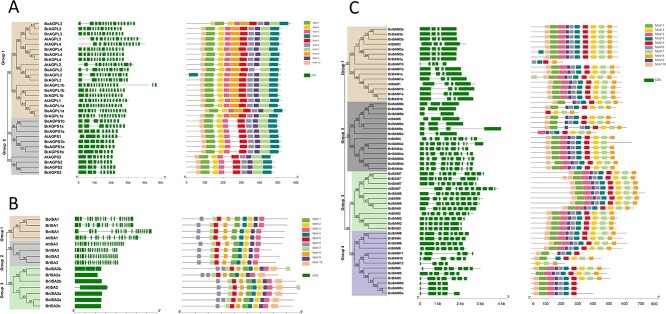
Phylogenetic relationships, structures, and conserved motifs of genes in the three families related to starch metabolism. **a**, **b** AGPase (**a**) and ISA (**b**) gene family related to the starch synthesis pathway. **c** BAM gene family related to the starch degradation pathway. The left part of each panel shows the phylogenetic tree, with different clades presented in different colors; the middle part shows exon–intron structures of genes, and green boxes represent untranslated 5′ and 3′ regions; the right part shows motif compositions of proteins; motifs 1–10 are displayed in different colored boxes.

Expression analysis revealed that among six gene families ISA, AGPase, and BAM family members are associated with the starch content of *P. brassicae*-infected roots in *B. rapa*. Therefore, we performed an analysis of the phylogenetic and structure of these three families. Eight genes encoding AGPases of the starch biosynthetic pathway were identified in the genome of *B. rapa*, which is similar to the number of AGPase genes in other Brassicaceae species (i.e. six in *A. thaliana* and eight in *B. nigra* and *B. oleracea*) (Table 1). Based on the phylogenetic relationships, 30 AGPase genes were divided into two subgroups, of which 19 AGPase genes encoded AGPL subunits and 11 encoded AGPS subunits. The AGPL subunits had more exons (13.89) than the AGPS subunits (8.18). Furthermore, motif 8 was only detected in the AGPL subunits (Fig. 6a). Four ISA genes were identified in *B. rapa*, *B. oleracea*, and *B. nigra*. According to the phylogenetic analysis, the ISA genes in the three *Brassica* species and *A. thaliana* were divided into three subgroups. The gene structure and motif analyses indicated that the genes in subgroup 3 had a simpler structure, with one exon, and included a unique motif (i.e. motif 10) (Fig. 6b). Fourteen BAM genes of the starch degradation pathway in *B. rapa*, 13 BAM genes in *B. nigra*, and 18 BAM genes in *B. oleracea* were identified based on their similarity to the *A. thaliana* BAM gene and the profile hidden Markov model (HMM). On the basis of the phylogenetic analysis, the BAM genes were divided into four groups (Fig. 6c). Accordingly, most BAM genes within a particular group had a similar exon–intron organization, which may reflect a common gene evolutionary process.

### Segmental duplication and synteny analysis of starch metabolism genes

Genome-wide duplication events are important for increasing genomic complexity during plant evolution. We identified the genome-wide collinear duplicated blocks in the *B. rapa* genome. More specifically, 18 of 38 starch metabolism genes (47.36%) were associated with segmental duplications (Supplementary Fig. S2, Supplementary Table S2), which were distributed on nine chromosomes. We identified four segmental duplication events involving eight genes in the BAM family, three segmental duplication events in the AGPase gene family, and one segmental duplication event in the SBE gene family as well as in the ISA gene family. There were no gene duplication events in the AMY and SS gene families. Duplicated gene pair *K*_a_/*K*_s_ ratios spanned from 0.03 to 0.20 (Supplementary Table S2), indicating that these genes evolved under negative selection pressure during the evolution of *B. rapa*. There were no tandem duplication events among the starch metabolism genes. These results indicate that segmental duplications were a major driver for the evolutionary expansion of the starch metabolism gene families in the *B. rapa* genome.

**Figure 7:**
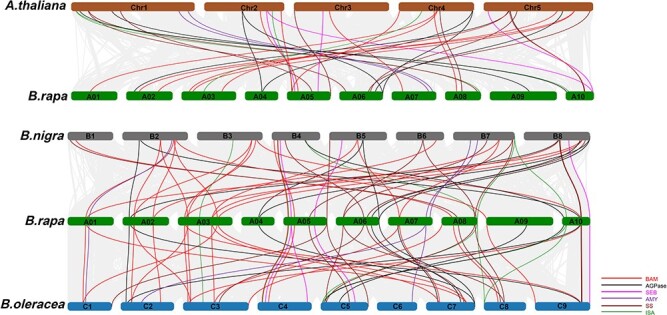
Syntenic relationships between *B. rapa* starch metabolism genes and *A. thaliana*, *B. oleracea*, and *B. nigra* genes.

To characterize the phylogenetic relationships between the starch metabolism genes, we performed a synteny analysis and found that a total of 164 pairs of starch metabolism genes had syntenic relationships between *B. rapa* and *A. thaliana* (41 pairs), *B. nigra* (59 pairs), and *B. oleracea* (64 pairs) (Fig. 7; Supplementary Table S3). Of the 41 pairs among *B. rapa* and *A. thaliana*, there were one and two copies of 19 and 18 starch metabolism genes, respectively, in *B. rapa*. There were 59 homologous gene pairs between *B. rapa* and *B. nigra*, of which one, two, and three copies of 16, 16, and 2 starch metabolism genes, respectively, were retained in *B. rapa*. Of the 64 gene pairs among *B. rapa* and *B. oleracea*, one, two, and three copies of 16, 18, and 3 starch metabolism genes, respectively, were included in the *B. rapa* genome. A comparison among *B. rapa* and the three additional species revealed that the AMY and SS family members were single-copy genes in *B. rapa*. Interestingly, all of the starch metabolism genes had orthologous genes in all other *Brassica* species, except for *BrBAM10* (Supplementary Table S4). Hence, the core starch metabolism genes appear to have been highly conserved during the evolution of these plant species.

The *K*_a_/*K*_s_ ratio is important in estimating selection pressure on genes during plant evolution. The analysis of *K*_a_/*K*_s_ ratios for orthologous genes among four species showed that the ratios of segmental duplications and all orthologous gene pairs were <1 (Supplementary Table S3), indicating that starch metabolism genes may have experienced strong purifying selection pressure during plant evolution.

## Discussion

There has been much study focusing on the resistance of Brassicaceae species to clubroot, but there are relatively few published reports describing the mechanism underlying *P. brassicae* pathogenicity*. P. brassicae*-induced changes in host anatomy have been investigated in *A. thaliana* and *Brassica* species [4, 11, 12, 22, 33]. Detecting *P. brassicae* in host roots by microscopy is challenging because of the lack of a specific staining method. Previous studies involving light microscopy and scanning electron microscopy revealed *P. brassicae*-induced starch accumulation in infected roots [21]. However, the young secondary plasmodia are not easily distinguished from starch grains [11]. To more precisely analyze *P. brassicae* development, host cells, and starch grains in infected Chinese cabbage roots, a dual fluorescent staining method was developed in this study. Starch grains are visible in infected and uninfected Chinese cabbage from 14 to 35 DAI. Significant starch accumulation was clearly observed in infected Chinese cabbage at 21 and 28 DAI by microscopy and starch quantification (Figs 3 and 4). This staining method enabled the examination of *P. brassicae* developmental stages as well as the changes to the cell walls and starch grains during infection. A new report on the refined life cycle of *P. brassicae* indicated that the resting spore-forming stage lasts from the resting sporangia plasmodium to the resting spore [4]. In our study various colors were observed in the *P. brassicae* resting spore-forming stage in *B. rapa* and *A. thaliana*, which may indicate that the cell wall or cytoplasm components of unmatured resting spores are different (Fig. 2). Starch grains were also observed in the isolated *P. brassicae* resting spores, which indicates the co-existence of undegraded starch grains in the mature stage with resting spores in host cells [12] (Fig. 1a). With the advantage of rapid and easy staining, and the ease of distinguishing host and *P. brassicae* cells, the dual fluorescent staining method will be very useful for further elucidating *P. brassicae* life stages as well as the anatomical changes in the host tissues.

### Correlation between starch content and *P. brassicae* infection

Exploring genomic commonalities may provide valuable information and clues for researchers on clubroot. Starch, as the predominant carbohydrate in plants, is synthesized in leaves and non-photosynthetic storage organs. Starch metabolism is a critical part of the plant life cycle partly because starch is a key molecule related to abiotic and biotic stresses [13, 34]. Based on a histochemical analysis, we found that *P. brassicae* infection of Chinese cabbage roots leads to an increase in the abundance of amylopectin*.* Several earlier studies proved that in response to abiotic stresses plants mobilize their starch reserves to release energy, sugar, and derived metabolites [13, 35, 36]. However, the effects of starch metabolism on biotic stress responses remain unclear. The starch content is reportedly positively correlated with the degree of infection in potato inoculated with the pathogenic fungus *Alternaria alternata* [14]. In *A. thaliana*, the starch contents are higher in roots infected by *P. brassicae* than in uninfected control roots [19]. In the current study, we observed that during *P. brassicae* infection the starch content increased sharply in the third week after inoculation and then decreased slowly. Similarly, in a recent study the starch content in olive plants infected by *Verticillium dahliae* decreased as disease symptoms developed [37]. Genome sequencing projects have revealed *P. brassicae*’s potential for manipulating plant metabolism to take up host nutrients [29, 30]*.* This study may explore some commonalities and provide clues for *P. brassicae* infection and clubroot development. The accumulation of starch grains in *B. rapa* roots infected by *P. brassicae* has been identified and associated with clubroot development, and previous research on potato and rapeseed also revealed that the starch content is higher in infected plants than healthy plants [14–16].

### Characteristics and evolution of starch metabolism genes in *Brassica* species

To further clarify the molecular basis of starch accumulation, members of six starch metabolism gene families were identified in Chinese cabbage and classified based on comparative genomics and phylogenetic relationships. By analyzing the homology among genes from *A. thaliana*, *B. rapa* (AA genome), *B. nigra* (BB genome), and *B. oleracea* (CC genome), we found that almost all genes related to starch metabolism in *B. rapa* have an ortholog in the other three species; the exception was *BrBAM10*. Most of the starch metabolism genes were single-copy genes with syntenic relationships among the *A. thaliana* and the A, B, C genomes. The exceptions were *AGPL1*, *AGPS1*, and *ISA2*, which were duplicated and homologous in *Brassica* species, implying the duplication events for these genes occurred after the *A. thaliana* lineage separated from the *Brassica* ancestor (43.2 million years ago) [38] and before the A, B, and C genomes diverged (7.9 million years ago) [39]. Additionally, there are more BAM genes in *Brassica* species than in *A. thaliana*. In *B. oleracea*, three genes (*BoBAM10*, *11*, and *12*), which are not homologous to *A. thaliana* genes, encode the glyco_hydro_14 domain and are considered to be members of the BAM gene family. This may be related to a base sequence change during evolution, which resulted in the neofunctionalization of genes. Finally, the *K*_a_/*K*_s_ ratios were all <1, indicating that these starch metabolism-related genes were under negative selection pressure during *B. rapa* evolution.

The genetic relationships among *B. rapa*, *B. nigra*, and *B. oleracea* can be explained by the triangle of U [40]. Furthermore, the model plant *A. thaliana* is a member of the family Brassicaceae and has a common ancestor with *Brassica* species. Therefore, in the current study, with reference to *A. thaliana* genes, we systematically analyzed the six core enzyme gene families related to starch metabolism in *Brassica* crops, with a specific focus on *B. rapa.* Comparative analysis of orthologous pairs of starch metabolism genes in the four species showed that 36 (97.30%) genes in *B. rapa* corresponded to genes in the genomes of *A. thaliana*, *B. nigra*, and *B. oleracea*. These results indicated starch metabolism genes were highly conserved in the four cruciferous species during evolution.

### Potential role of starch metabolism genes in the response of *B. rapa* to *P. brassicae*

Previous transcriptomics-based analyses of *Brassica* crops identified several differentially expressed genes associated with the starch metabolic pathway that may influence plant responses to *P. brassicae* [20, 41–43]. Another study revealed that during *P. brassicae* infection of *A. thaliana*, SS genes are more highly expressed at 10 days after inoculation than at 23 days after inoculation [44]. In the current study, the expression levels of genes related to the starch synthesis pathway, especially *BrAGPS2* and *BrISA2b*, were significantly up-regulated at 7 DAI with *P. brassicae*. The expression levels of starch degradation-related genes were significantly up-regulated at 14 DAI, including *BrBAM4a*, *BrBAM4b*, and *BrBAM8*. The expression of these starch metabolism genes was positively correlated with the starch content.

Based on our results, we propose that the accumulated starch (amylopectin) provides *P. brassicae* with carbon and energy during the infection of *B. rapa*. After the host plant perceives the infection, specific genes related to starch synthesis are activated, leading to an increase in starch contents. When the infected plant detects the proliferation of *P. brassicae*, the genes related to starch degradation are activated to provide the pathogen with carbon and energy, which are used for proliferation. We observed that the expression patterns of two starch synthesis pathway genes (*BrAGPS2* and *BrISA2b*) and three starch degradation pathway genes (*BrBAM4a*, *BrBAM4b*, and *BrBAM8*) were consistent with the starch contents. Therefore, we believe these five genes are the key genes mediating the response of *B. rapa* to *P. brassicae*. The findings of this research provide novel insights into the relationships among the core genes involved in starch metabolism in *B. rapa* infected by *P. brassicae*.

### Conclusions

In this study, we developed a dual fluorescent staining method applicable for distinguishing between *P. brassicae*, cell structures, and starch grains in infected Chinese cabbage. By combining the results of cytological and starch quantification analyses, we demonstrated that the starch content significantly increased from 14 to 21 days after the *P. brassicae* infection was initiated. Moreover, 38 genes encoding core enzymes contributing to starch metabolism were identified based on genome-wide analysis. The expression levels of genes participating in the starch synthesis pathway increased significantly from 7 to 14 days after plants were inoculated with *P. brassicae*. The expression of genes involved in starch degradation increased significantly after 14 days. Finally, according to syntenic analysis, the starch metabolism genes were revealed to be highly conserved in *A. thaliana*, *B. rapa*, *B. nigra*, and *B. oleracea.* The data presented herein may be useful for characterizing the evolution and regulatory effects of the core starch metabolism genes in Chinese cabbage. This study provides new insights into *P. brassicae* pathogenesis and interactions with *Brassica* plant hosts.

## Materials and methods

### Plant materials

The Chinese cabbage inbred line ‘BJN3-1’ and an *A. thaliana* ecotype Columbia (Col-0) were used in this study. ‘BJN3-1’ is highly susceptible to *P. brassicae* [45].

### Pathogen inoculation

*P. brassicae* isolate ‘LNXM-8’, which was identified as pathotype Pb1 according to the Sinitic clubroot differential set, was used to inoculate ‘BJN3-1’ and Col-0 plants [45]. The preparation of resting spores and the inoculation process followed Pang *et al.* [45]. ‘BJN3-1’ plants treated with distilled water were used as the mock control.

### Dual fluorescent staining and examination

The middle part of the main root of Chinese cabbage ‘BJN3-1’ and Col-0 plants was washed and placed in a 10% formalin solution used for preparing paraffin sections (Supplementary Fig. S4). Root samples of ‘BJN3-1’ were collected at 7, 14, 21, 28, and 35 DAI with *P. brassicae* or distilled water. Root samples of Col-0 were collected at 28 DAI with *P. brassicae*. Ten repeats of root cross-sections were prepared as previously described [46]. Paraffin sections were stained with 0.05% aniline blue for 5 minutes and then washed with distilled water and ethanol for 30 seconds each. The aniline blue-stained samples were then stained with 10 μg/ml Nile red (prepared in acetone) for 60 seconds and then washed with ethanol and distilled water for 30 seconds. Slides were examined and images were captured using an IX83 fluorescence microscope (Olympus, Tokyo, Japan).

### Starch detection and determination

Chinese cabbage roots inoculated with *P. brassicae* or distilled water were collected at 3, 7, 14, 21, 28, and 35 DAI. Three replicates of 20 individual plants were used for quantifying starch using a commercial starch assay kit (Comin Company, Suzhou, China). For a qualitative analysis of starch, 10 repeat root cross sections prepared from samples collected at 21 DAI along with amylose and amylopectin from potato were stained with a fresh iodine solution (I_2_/KI) for 3 minutes and then washed with distilled water for 30 seconds.

### Identification of core genes related to starch metabolism

Sequences of the annotated *A. thaliana* genome were retrieved from the TAIR10 database (https://www.arabidopsis.org/). Additionally, *B. rapa*, *B. nigra*, and *B. oleracea* genome assembly and gene annotation data were downloaded from the BRAD database (http://brassicadb.cn/). We identified the starch metabolism genes according to sequence similarities and specific profile HMMs. To determine sequence similarities, a BLASTP analysis was used to identify the predicted starch metabolism genes, using *A. thaliana* genes as queries and the following criteria: e-value <1e−20, identity >70%, and match length > 70% (of the shorter protein sequence). Regarding the profile HMM approach, domains in the *A. thaliana* starch metabolism genes were detected according to the Pfam 33 database. We then used hmmsearch of the HMMER program (version 3.3) [47] and the HMM analogous to the specific domains in Pfam to screen for and identify starch metabolism genes in the *B. rapa*, *B. nigra*, and *B. oleracea* genomes. We confirmed the homologous relationships among these starch metabolism genes. Multiple gene sequences were aligned using CLUSTALW [48] and phylogenetic trees were created according to the neighbor-joining method of MEGA-X (with 1000 bootstrap replicates) [49]. Finally, starch metabolism genes were manually screened on the basis of the phylogenetic trees and sequence properties.

### Total RNA extraction and analysis of gene expression

Entire roots were sampled at 3, 7, 14, 21, 28, and 35 DAI with *P. brassicae* or distilled water. Three replicates of 20 individual plants were used for the subsequent RNA isolation as well as qRT–PCR analysis. Firstly, total RNA was extracted from infected and mock control ‘BJN3-1’ root samples using TRIzol™ reagent (Invitrogen, Carlsbad, USA). Subsequently, cDNA was synthesized from the purified total RNA (2 μg) using SuperScript™ III Reverse Transcriptase (Invitrogen, Carlsbad, USA). The Primer 3.0 online program was used to design the primers for qRT–PCR; the primer information is listed in Supplementary Table S5. The qRT–PCR was conducted in SYBR Green Supermix (Bio-Rad Company, Hercules, USA) with the CFX96™ Real-Time System. All experiments were performed in three biological replicates. The relative expression levels of the genes were determined by the 2^−∆∆Ct^ method [50], and the sample from each time point for distilled water treatment was used as the control.

### Chromosomal position and duplication events of starch metabolism genes

All identified starch metabolism genes were mapped to the *B. rapa* chromosomes based on physical location in the reference genome and TBtools software [51] was used for drawing the map. The duplication events of the starch metabolism genes in *B. rapa* were detected based on MCScanX [52] with default parameters. Then, the tandem duplication genes were determined based on their physical position, with no more than one intervening gene. The *K*_a_/*K*_s_ ratios of the duplication events were calculated by KaKs_Calculator (version 2.0) [53].

### Analysis of orthologous gene pairs among *A. thaliana*, *B. rapa*, *B. nigra,* and *B. oleracea* genomes

In this study, we use MCScanX to detected orthologous gene events between *B. rapa* and the other three species (*A. thaliana*, *B. nigra*, and *B. oleracea*). The parameters were as follows: *e* = 1*e*−20, *u* = 1, and *s* = 5 [54]. TBtools was used to extract orthologous pairs of starch metabolism genes and maps for assessing synteny.

## Acknowledgements

This study received funds from the Liaoning Natural Science Foundation (2021-MS-229), Liaoning Revitalization Talents Program (XLYC1807242), the Key Research and Development Program of Hubei province, China (grant numbers 2020BBB083 and 2020BBA037), the Korea Institute of Planning and Evaluation for Technology in Food, Agriculture, Forestry and Fisheries through the Golden Seed Project, which is funded by the Ministry of Agriculture, Food and Rural Affairs (213006-05-5-SB110). These funding organizations had no role in the planning of this study, collection, analysis, interpretation of the data, and in the writing of the manuscript.

## Author contributions

Y.W., Y.B.M., and W.X.P. carried out the experiments and generated the data. Y.B.M., SRC, and WXP analyzed all data and wrote the original manuscript. X.Z., Y.J.W., X.Y.Z., M.Y.Z., D.L., Z.N.Z., D.Z., and X.Y.Y. participated in plant growth and dual fluorescent staining method development. C.X.G., S.S.C., and Y.L. participated in writing and modifying the manuscript. W.X.P., Y.P.L., and Y.L. conceived the study, participated in its coordination, and corrected and modified the manuscript. All authors have read and approved the final manuscript.

## Data availability

The data generated herein to support the results of this study are presented in the paper and the supplementary information files. The data involved in the phenotypic study are accessible from a corresponding author upon request. The *Arabidopsis* and *Brassica* genome databases used to generate data of this study are available in TAIR (https://www.arabidopsis.org/) and BRAD (http://brassicadb.cn), respectively.

## Conflict of interest

The authors declare no competing interests.

## Supplementary data

Supplementary data is available at *Horticulture Research* online.

## Supplementary Material

Web_Material_uhab071
